# Role of High-Sensitivity C-reactive Protein Levels in Predicting the Risk of Six-Month Event Rates in Patients With Chronic Stable Angina Undergoing Percutaneous Transluminal Coronary Angioplasty With a Drug-Eluting Stent

**DOI:** 10.7759/cureus.38457

**Published:** 2023-05-02

**Authors:** Ranjit K Nath, Dheerendra Kuber, Puneet Aggarwal, Shivani Rao

**Affiliations:** 1 Cardiology, Atal Bihari Vajpayee Institute of Medical Sciences (ABVIMS) and Dr. Ram Manohar Lohia (RML) Hospital, New Delhi, IND

**Keywords:** pci (percutaneous coronary intervention), high-sensitivity c-reactive protein, chronic stable angina, drug-eluting stent, early ptca (percutaneous transluminal coronary angioplasty)

## Abstract

Introduction

This prospective observational study reports the association between baseline high-sensitivity C-reactive protein (hs-CRP) levels and adverse events at six months in patients who were diagnosed with symptomatic chronic stable angina and then underwent percutaneous transluminal coronary angioplasty (PTCA) with a drug-eluting stent (DES).

Methods

A total of 104 patients were examined with chronic stable angina over a period of six months. Before conducting percutaneous coronary intervention (PCI), the baseline levels of hs-CRP were measured, and based on the levels, the patients were grouped into high and low hs-CRP groups.

Results

The primary causes of death or the need for repeat revascularization or myocardial infarction or angina were concluded after assessing the patients for six months. A total of 104 patients were studied, among which 72 (69.23%) had low hs-CRP and 32 (30.77%) had high hs-CRP levels. The number of males in this study was 68 (65.38%) and females were 36 (34.62%). The mean age of the patients was 55.26 ± 10.31 years. There were no significant differences among the groups in terms of gender, age, comorbidities, and risk factors except for certain predisposing factors like dyslipidemia and smoking. Moreover, we did not find any significant difference among the groups in the cause of death and myocardial infarction after a follow-up of six months. However, we observed a higher need for revascularization and angina outcomes in the group with high hs-CRP compared to low hs-CRP.

Conclusion

It can be concluded that a higher risk of angina and repeat revascularization is related to a high baseline hs-CRP but there is no evidence whether it is somehow linked to myocardial infarction and mortality or not.

## Introduction

Coronary artery disease (CAD) is one of the major causes of morbidity and death in industrialized and developing countries [1,2]. The pathogenesis of both plaque instability and atherosclerosis is closely related to inflammation. C-reactive protein (CRP) is an important marker for cardiovascular events [3] but is not specific for systemic inflammation [4]. Increased CRP levels affect the complement pathway, leading to adverse coronary events associated with endothelial dysfunction [5]. Initially, the traditional assays were not much sensitive to detect the levels of CRP for the prediction of cardiovascular diseases. However, recently developed CRP assays are very sensitive, reducing the problem [6]. It has been previously reported that in comparison to <1 mg/l hs-CRP levels, >3 mg/l was related to a 60% higher risk of coronary heart disease (CHD) incidence after adjustment of all Framingham risk variables (RR: 1.60; 95% CI: 1.43 to 1.78) [7]. Recent studies in patients with percutaneous coronary revascularization have also reported that increased baseline inflammatory levels are associated with adverse outcomes [8]. We studied the role of hs-CRP as an independent inflammatory marker and short-term predictor of adverse events in patients who have undergone percutaneous coronary revascularization in this study.

## Materials and methods

This prospective observational study was conducted for a period of one year in the department of cardiology in a tertiary care center at Atal Bihari Vajpayee Institute of Medical Sciences (ABVIMS) and Dr. Ram Manohar Lohia (RML) Hospital, New Delhi. The inclusion criteria for this study were patients with symptomatic chronic stable angina who underwent percutaneous transluminal coronary angioplasty (PTCA) with a drug-eluting stent. Informed consent was obtained from all patients. The exclusion criteria included patients with a history of prior revascularization, acute coronary syndrome (unstable angina (USA), ST-elevation myocardial infarction (STEMI), and non-STEMI (NSTEMI)), with current infection, on current treatment for malignancy, with acute stroke or acute limb ischemia, and usage of immunosuppressive drugs. Prior to intervention, hs-CRP levels were measured in fasting blood samples by using an enzyme-linked immunosorbent assay (ELISA) kit called a Calbio kit (Calbiotech, El Cajon, CA) with a sensitivity range of 0.01 to 10 mg/l. The enrolled patients underwent PTCA with a drug-eluting stent (DES). They were subjected to complete revascularization with percutaneous coronary intervention (PCI) in all major arteries. All patients received optimal medical therapy based on the evidence. The clinical follow-ups were sincerely done at two weeks, one month, three months, and six months. During the six-month study duration, all the major events, such as myocardial infarction, repeat revascularization, angina, and mortality, were recorded as endpoints.

## Results

There were a total of 123 patients enrolled in this study. But the results of the study were based on 104 patients since 19 patients lost follow-up. The patients enrolled for the study were divided into two groups based on the median levels of CRP i.e., high CRP levels >3 mg/l and low CRP levels <3 mg/l. Out of 104 patients, 32 (30.77%) were placed in the high hs-CRP group and 72 (69.23%) were in the low hs-CRP group (Table 1).

**Table 1:** Group distribution hs-CRP: high-sensitivity C-reactive protein

hs-CRP (mg/l)	Frequency	Percentage
<3	72	69.23%
>3	32	30.77%
Total	104	100.00%

 The age of the patients ranged between 30 years and 80 years with a mean age of 55.26 ± 10.31 years (Table 2).

**Table 2:** Age distribution

	Sample size	Median	min	Max	Mean ± STD
Age (years)	104	56	30	80	55.26 ± 10.31

A total of 68 (65.38%) male and 36 (34.62%) female patients were studied (Table 3).

**Table 3:** Gender distribution

	Frequency	Percentage
Female	36	34.62%
Male	68	65.38%
Total	104	100.00%

 There was no significant difference observed in terms of gender (P=>0.63) and age (P=>0.54) between the two groups (Tables 4, 5).

**Table 4:** Age distribution among two groups hs-CRP: high-sensitivity C-reactive protein

	Baseline hs-CRP ( mg/l)		
	<3	>3	P value
Age (mean in years )	54.17	57.72	0.54
No of patients	72	32	

**Table 5:** Gender distribution among the two groups hs-CRP: high-sensitivity C-reactive protein

		Baseline hs-CRP (mg/l)		Total	P value
		<3	>3		
Gender	F	26 (36.11%)	10 (31.25%)	36 (34.62%)	0.631
	M	46 (63.89%)	22 (68.75%)	68 (65.38%)	
Total		72 (100.00%)	32 (100.00%)	104 (100.00%)	

We also studied various risk factors and comorbidities, including hypertension, diabetes, smoking, dyslipidemia, and diseased vessel and echocardiographic left ventricular ejection fraction in both groups. It was found that there was a statistically significant difference in dyslipidemia (P=>0.017) and smoking (P=>0.005) between the two groups (Tables 6, 7).

**Table 6:** Dyslipidemia among the groups hs-CRP: high-sensitivity C-reactive protein

		Baseline hs-CRP(mg/l)		Total	P value
		<3	>3		
Dyslipidemia	No	43 (59.72%)	11 (34.38%)	54 (51.92%)	0.017
	Yes	29 (40.28%)	21 (65.63%)	50 (48.08%)	
Total		72 (100.00%)	32 (100.00%)	104 (100.00%)	

**Table 7:** Smoking among the groups hs-CRP: high-sensitivity C-reactive protein

		Baseline hs-CRP (mg/l)		Total	P value
		<3	>3		
Smoking	No	52 (72.22%)	14 (43.75%)	66 (63.46%)	0.005
	Yes	20 (27.78%)	18 (56.25%)	38 (36.54%)	
Total		72 (100.00%)	32 (100.00%)	104 (100.00%)	

All the enrolled patients had a normal range of hemoglobin and kidney function test levels. In each group, one death was reported in this study due to sudden cardiac arrest leading to deaths. Though no particular cause of death was identified. We did not observe any statistically significant difference between the two groups (P=>0.523). Angina was reported in seven (6.73%) patients. The number of angina patients in the high hs-CRP group, i.e. five (15.63%) was significantly higher than the low hs-CRP group, i.e. two (2.78%) (P=>0.027) (Table 8).

**Table 8:** Chronic stable angina in six months hs-CRP: high-sensitivity C-reactive protein

		Baseline hs-CRP (mg/l)		Total	P value
		<3	>3		
Stable angina	No	70 (97.22%)	27 (84.38%)	97 (93.27%)	0.027
	Yes	2 (2.78%)	5 (15.63%)	7 (6.73%)	
Total		72 (100.00%)	32 (100.00%)	104 (100.00%)	

Post the six-month follow-up, myocardial infarction was reported in four (3.85%) patients in total, out of which one (1.39%) was in the low hs-CRP group and three (9.38%) were in the high hs-CRP group, though this is not statistically significant (P=>0.085). Six (18.75%) patients in the high hs-CRP group and two (2.78%) patients in the low hs-CRP group underwent repeat revascularization during the period of six months of follow-up, which was statistically significant (P=>0.010) (Table 9).

**Table 9:** Need for repeat revascularization hs-CRP: high-sensitivity C-reactive protein

		Baseline hs-CRP (mg/l)		Total	P value
		<3	>3		
Repeat revascularization	No	70 (97.22%)	26 (81.25%)	96 (92.31%)	0.010
	Yes	2 (2.78%)	6 (18.75%)	8 (7.69%)	
Total		72 (100.00%)	32 (100.00%)	104 (100.00%)	

## Discussion

We studied 104 patients with chronic stable angina. Complete revascularization by PCI and drug-eluting stents was done in all patients enrolled in this study. No statistically significant differences were found in association with risk factors, age, and comorbidities of hypertension and diabetes except for dyslipidemia and smoking among both groups. There were 38 (36.54%) smokers in this study, which included 18 (56.25%) in the high hs-CRP group and 20 (27.78%) in the low hs-CRP group (P =>0.005). This clearly highlights that patients with a history of smoking had high baseline levels of hs-CRP. A similar conclusion was also reported by de Winter RJ et al., where they found the smokers in the study had an increased CRP level of >3 mg/L and were mostly elderly women [9]. Another study by Gallus et al. has shown that cessation of smoking is associated with a decrease in hs-CRP levels, which again indicates smoking is directly proportional to increased hs-CRP levels. They observed that the prevalence of baseline CRP levels of current smokers were 41.1%, whereas that of former smokers was 35.8% [10]. Similar findings were also observed by Cho JH et al., who observed the duration of smoking cessation was significantly more in the non-elevated hs-CRP group, whereas the participants who did not quit smoking had elevated CRP levels [11]. However, these patients had predisposing factors like diabetes or hypertension, which contradicts our findings. We had 50 (48.08%) patients with dyslipidemia in our study. According to Cho JH et al., it was noticed that 65% of these patients had high CRP levels and similar findings were also reported elsewhere showing that baseline dyslipidemia is linked to high hs-CRP levels [12]. We studied the events caused in six months, which included repeat revascularization, acute coronary syndrome, and chronic stable angina. Of 104 patients, 13 (12.5%) patients were found to suffer any event during the six-month follow-up period.

Chronic stable angina was reported in seven (6.73%) patients, acute coronary syndrome (STEMI, NSTEMI, USA) in four (3.8%) patients, and death in two (1.92%) patients during follow-up. We evaluated the seven patients with symptoms of chronic stable angina on the treadmill and found five out of seven had high baseline levels of hs-CRP and the levels of the other two patients were normal. This gives a clear indication of the association of high baseline hs-CRP levels with the risk of chronic stable angina. In another study by Antonino Buffon et al., 219 consecutive patients were studied who underwent PTCA on a single non-occlusive coronary stenosis [13]. They reported 29% of patients showed increased levels of CRP and 40% of patients underwent clinical restenosis and hence concluded that the percentage of clinical restenosis was significantly more in patients with increased CRP levels. Further, a study by Rahel BM et al. reported that the recurrence of complaints for angina was found in 32.7% of patients in a follow-up period of eight months [14]. Similar outcomes were also seen in the current study, where 16.12% out of 32 patients with high levels of baseline hs-CRP came back with recurrent angina. It was also found in the study that increased baseline hs-CRP levels and increased risk of chronic stable angina were strongly associated with baseline diffuse coronary artery diseases. Hence, it can be concluded that the risk of recurrent chronic stable angina after PCI is higher in patients with increased baseline hs-CRP levels. In a study by de Winter RJ et al., during follow-up, it was noticed that there is an increased risk of myocardial infarction in patients with elevated levels of hs-CRP. The percentage of myocardial infarction was 23 (3.2%) in the high CRP group compared to six (0.8%) in the normal group [9]. On the other hand, another study by Rahel et al. reported that during the follow-up for eight months, there were just 54 (9%) patients with a major adverse clinical event (repeat PCI: 57%, myocardial infarction: 23%, coronary artery bypass graft (CABG): 13%, death: 7%, all deaths were cardiac or presumed to be cardiac). Though not statistically significant, the levels of CRP were higher in patients with major adverse cardiovascular events (MACE) as compared to those without MACE. Though statistically insignificant, there were three (9.38%) patients from the high hs-CRP group and one (1.39%) patient from the low hs-CRP group, making a total of four (3.85%) patients with ACS in this current study. Another study by Shitara et al. reported that in a prospective study of 3507 consecutive CAD patients, elevated levels of CRP during the follow-up of angiography were related to higher chances of all-cause death and ACS (ACS 2.14, 95% confidence interval, p = 0.0002) [15]. This indicates that there is no association of inflammatory markers like hs-CRP with the occurrence of acute events in a short-term follow-up study. For further confirmation, there is a need for a randomized study with a larger sample size and a longer term of follow-up.

In this study, there were eight (7.69 %) patients who underwent repeat revascularization at the time of the six months follow-up. Six (18.75%) patients out of 32 with high levels of hs-CRP underwent revascularization compared to only two (2.78%) out of 72 in the low hs-CRP group. The data obtained were statistically significant (P=>0.010), thus indicating that a higher rate of revascularization is associated with high baseline levels of hs-CRP in patients. A similar outcome was also reported by de Winter RJ et al. who found patients with elevated levels of hs-CRP had an increased rate of revascularization, though the data was statistically insignificant [9]. The aim of our study was not to find the cause of the lesion for revascularization, i.e. whether it is tricuspid valve repair (TVR) or lesion revascularization (TLR). It was reported in previous literature that TLR, in the form of in-stent restenosis (ISR) is required by patients with chronic stable angina; however, in the case of ACS, newer culprit lesions were responsible. Another study by Walter DH et al. found that when the patients were grouped into tertiles based on pre-procedural CRP levels, it was seen that though baseline angiographic and clinical characteristics were identical and after implantation of a stent, a primary endpoint event was reported in 24 (26%) patients of the lowest tertile, in 42 (45.6%) of the middle tertile and in 38 (41.3%) of the highest CRP tertile (P< 0.01) [16]. It was found that the CRP levels tertiles were associated independently with increased chances of adverse coronary events (relative risk = 2.0 (1.1 to 3.5), tertile I vs. II and III, P< 0.01) along with minimal lumen diameter post stent (P< 0.04). Moreover, it was shown that the rate of restenosis was significantly more in the upper two tertiles in comparison to levels of CRP in the lowest tertile (45.5% vs. 38.3% vs. 18.5%, respectively, P< 0.002). Moreover, there was another study by Kyeong Ho Yun et al. on consecutive 360 patients undergoing elective coronary stenting. In this study, inflammatory response to PCI was calculated by taking the difference between the peak post-procedural hs-CRP levels and the pre-procedural hs-CRP levels, and a significant association was found between CRP and the changes in troponin T after PCI (rr=0.210, P<0.001). The prediction for the incidence of MACE was higher in the level of hs-CRP >3 mg/l compared to low hs-CRP (hazard ratio 2.1, P<0.005) concluding that hs-CRP elevation >3 mg/l after the procedure is linked to the increased chances of MACE in patients with ACS. CRP determinations may be of value for risk stratification after PCI. Luo S et al. have shown that increased baseline CRP level is an independent predictor of cardiovascular mortality, MACE, and all-cause mortality in CAD patients [17]. Thus, further validation is needed to prove that the baseline hs-CRP levels are associated with the risk of repeat revascularization.

A study on 1458 consecutive patients who underwent elective coronary angioplasty for Braunwald class I/II/III angina was conducted by de Winter RJ, where the patients were followed up for 12 to 14 months [9]. In this study, there were 21 (2.9%) deaths in the high CRP group in comparison to five (0.7%) in the normal CRP group (P=0.002). It was concluded from this study that a post-coronary angioplasty, elevated level of CRP is an independent prognostic indicator of death, contrary to the findings of our study where we observed two sudden cardiac deaths, one in each group. We could not evaluate the proper cause of death since the deaths occurred outside the hospital. We did not find any statistical significance between the two groups in terms of death during the six-month follow-up. Thus, we cannot conclude the statement “Increased CRP levels are an independent prognostic indicator for the occurrence of death.”

Limitations of the study 

This was a single-center study with a small sample size. Multiple confounding factors were not addressed systematically and then impacts on outcomes were not evaluated, for example, smoking, dyslipidemias, diabetic control, hypertension control, and drug compliance. However, in patients with multi-vessel PCI, we could not exclude the effects of "one-sitting PCI vs staged PCI". Recently, there is a controversy about which procedure is superior, one-sitting PCI or staged PCI in multi-vessel disease.

## Conclusions

Echoing the findings of previous studies, we conclude that higher levels of hs-CRP pose a risk for recurrent event rate and predicting acute events in the future. Further studies are required with a larger sample size, longer follow-up, and the inclusion of various factors to build a concrete conclusion.
